# From screening to follow-up: a review of quality of life and psychological status across the clinical care continuum in patients with differentiated thyroid cancer

**DOI:** 10.3389/fpsyg.2025.1721117

**Published:** 2025-12-05

**Authors:** Zifeng Kuang, Hao Zhao, Chenjia Zhang, Xiaoyi Li

**Affiliations:** 1 Department of General Surgery, Peking Union Medical College Hospital, Chinese Academy of Medical Sciences & Peking Union Medical College, Beijing, China; 2 Surgery Centre of Diabetes and Mellitus, Beijing Shijitan Hospital, Capital Medical University, Beijing, China

**Keywords:** differentiated thyroid cancer, quality of life, mental health, clinical care continuum, thyroid nodule

## Abstract

**Background:**

The global incidence of differentiated thyroid cancer (DTC) has risen rapidly in recent years. Given its favorable prognosis, extended survival, and expanding patient population, the quality of life (QOL) and psychological wellbeing of patients have gained importance in DTC management.

**Methods:**

This narrative review summarizes recent research on the QOL and psychological health of DTC patients across all stages of clinical care continuum, including screening, diagnosis, treatment, and follow-up.

**Results:**

DTC patients commonly experience impaired QOL and psychological distress throughout the clinical care continuum. Thyroid screening and nodules detection often trigger anxiety and depression, although their severity and the need for medical intervention remain unclear. Patients undergoing fine-needle aspiration frequently experience anxiety regarding the potential diagnosis of malignancy. Even those diagnosed with benign nodules may experience anxiety due to the need for long-term surveillance and the uncertainty associated with the process. While patients with low-risk DTC managed by active surveillance generally report better QOL than those who undergo surgery, concerns regarding disease progression persist. Psychological factors significantly influence treatment decision-making. Conventional surgery can lead to adverse events that reduce QOL and increase psychological burden, while minimally invasive approaches may reduce scar-related concern but offer limited improvement in overall QOL. Furthermore, hypothyroidism prior to radioactive iodine therapy and the treatment-related adverse effects often lead to a transient decline in wellbeing, whereas the effects of TSH suppression therapy remain uncertain. Although many of these negative effects can resolve over time, DTC patients continue to report worse QOL and psychological wellbeing compared to the general population, which can be attributed to enduring fears of recurrence and insufficient informational and emotional support.

**Conclusion:**

DTC patients encounter significant challenges related to QOL and psychological health at all stages of management. There is a pressing need for comprehensive care and supportive interventions throughout the clinical continuum to enhance the overall health of DTC patients.

## 1 Introduction

The global incidence of thyroid cancer has increased markedly in recent years. In 2022, over 821,000 new cases of thyroid cancer were diagnosed worldwide, making it the seventh most common malignancy and one of the fastest-growing cancers by incidence (Bray et al., 2024). Notably, differentiated thyroid cancer (DTC) accounts for approximately 90% of all thyroid cancer cases (Boucai et al., 2024). Standard treatment for DTC typically includes surgery, radioactive iodine (RAI) therapy, and long-term TSH suppression or replacement therapy. In recent years, for patients with papillary thyroid microcarcinoma ( ≤ 1 cm in diameter) without high-risk features, active surveillance (AS) has also been increasingly recommended as an initial management strategy (Ringel et al., 2025). Most DTC tumors grow slowly and have relatively indolent biological behavior, with 10-year post-treatment survival rate reaching 97.3% (Wilhelm et al., 2023). However, despite the high incidence and favorable prognosis, the majority of DTC patients experience a variety of physical and psychological challenges throughout the processes of screening, diagnosis, treatment, and follow-up, often resulting in significantly diminished quality of life (QOL) and mental health (Xiong et al., 2025). Therefore, it is critical to comprehensively assess the QOL and psychological wellbeing of DTC patients at each stage of disease management, and develop tailored interventions for optimizing treatment outcomes.

## 2 Methods

The recent literature on the QOL and psychological health of DTC patients has been systematically analyzed in this narrative review. The relevant original studies, systematic reviews, and meta-analyses were identified using terms related to thyroid cancer and patient-reported outcomes, and the current status and main challenges related to the QOL and psychological health of DTC patients throughout the clinical care continuum were summarized. The goal of this review is to provide a theoretical foundation for optimizing the management of DTC patients and guide future research. This review article does not involve any original research with human participants or identifiable data. According to the policy of the Ethics Review Committee of Peking Union Medical College Hospital, the study was exempt from ethical review. Informed consent was not applicable.

## 3 Psychological status during the screening and diagnostic phase of thyroid cancer

### 3.1 The psychological impact of thyroid nodule screening

Recent studies have shown that thyroid nodule screening can adversely affect the psychological wellbeing of examinees. Li et al. conducted a study on 2,834 individuals undergoing first-time thyroid nodules screening, and found that post-screening scores for anxiety, depression, and sleep disturbance were significantly worse than the respective pre-screening scores. Moreover, individuals who received a positive diagnosis of nodules (*n* = 1,153) had significantly worse scores compared to those without nodules (*n* = 1,681) (Li et al., 2021). Similar results have been reported in other studies (Yin et al., 2019; Jiang, 2017). In a large-scale retrospective study conducted by Kornelius et al. (2025) (*n* =138,803 pairs), patients with thyroid nodules had a higher risk of developing anxiety (HR 1.36; 95% CI: 1.34–1.38) and mood disorders (HR 1.16; 95% CI: 1.14–1.18) compared to the general population. These findings suggest that the screening procedure, as well as the subsequent detection of thyroid nodules may contribute to negative psychological outcomes.

However, the necessity of clinical intervention for these psychological issues remains uncertain. In an age-and sex-matched study, Yin et al. reported that among individuals with newly detected nodules (*n* = 306), 35.3% met the criteria for anxiety and 47.7% for depression, with 23.9% and 36.6% of the cases classified as mild, and 11.4% and 11.1% classified as moderate or above, respectively (Yin et al., 2019). However, using the same assessment tools and criteria, Jiang (2017) found that the rates of anxiety and depression among patients with thyroid nodules (*n* = 207) were only 10.6% and 13%, respectively. Furthermore, in the aforementioned study of Li et al., the mean scores for anxiety, depression and sleep disturbance in patients diagnosed with thyroid nodules remained below the clinical diagnostic cutoffs (Kornelius et al., 2025). Thus, the clinical significance of the psychological impact associated with thyroid nodule screening remains unclear and requires further research.

### 3.2 The psychological impact of thyroid cancer diagnosis

Patients with thyroid nodules frequently experience anxiety stemming from the uncertainty regarding their diagnosis, prompting many to pursue pathological confirmation through biopsy (Rosser, 2019; Jensen et al., 2020). Studies show that prior to thyroid fine-needle aspiration (FNA), approximately one-quarter of the patients exhibit psychological distress such as depression, anxiety, and insomnia (Zhu et al., 2023), and this proportion is significantly higher than that observed for healthy controls (20.31% vs. 4.69%, *p* = 0.007) (Moon et al., 2015). The pre-procedural anxiety may be related to the uncertainty regarding diagnostic outcomes, particularly concerns about a potential malignant diagnosis (Moon et al., 2015; Liu L. et al., 2024; Miller et al., 2014). Additionally, while some studies suggest that previous FNA experience can alleviate anxiety (Senoymak et al., 2024), others report a positive correlation between the number of FNAs and the prevalence of pre-procedural anxiety (4.69% in those with no prior FNA, 15.79% with one FNA, and 26.92% with multiple FNAs), which can be attributed to increased concerns about the possibility of a malignant diagnosis with each FNA (Moon et al., 2015). Furthermore, an insufficient understanding of the FNA procedure may exacerbate psychological stress. Previous studies have demonstrated that providing only written or verbal information is often insufficient to reduce pre-procedural anxiety (Jlala et al., 2010). Ay et al. (2022) showed that supplementing standard written information with video-based explanations significantly improved the pre-FNA anxiety scores (*p* = 0.003), whereas written information alone had no such effect. These findings suggest that providing clear information prior to the procedure may help alleviate patient anxiety.

Despite the generally favorable prognosis of thyroid cancer, many patients continue to experience negative emotional responses following a positive diagnosis. Pitt et al. conducted semi-structured interviews with 85 patients who underwent FNA and found that, regardless of whether the pathology was “indeterminate” or “malignant,” most patients experienced immediate emotional reactions such as anxiety, fear, and shock. Some described the cancer diagnosis as a major disruption and a life-altering event until surgery was completed (Pitt et al., 2021b). In a recent study assessing the pre-treatment status of newly diagnosed thyroid cancer patients, 33.67% exhibited significant psychological distress that require clinical intervention, with 30.3% and 17.3% experiencing fear and anxiety, respectively. Worries about disease recurrence (70.33%) and postoperative scarring (50.67%) were the main factors contributing to psychological distress (Lei et al., 2021). These findings indicate that the psychological burden in thyroid cancer patients arises not only from uncertainty about prognosis, but also from apprehensions regarding future QOL.

Although a clinical or pathological diagnosis of benign nodules may partially relieve the psychological burden on patients (Li et al., 2021), it does not necessarily result in better mental health compared to that following a malignant diagnosis. Kornelius et al. (2025) found that patients with thyroid nodules had a significantly higher risk of developing anxiety disorder than those with thyroid cancer (HR 1.06, 95% CI: 1.03–1.08), and this difference persisted over a 10-year follow-up period. This may be attributed to the need for long-term and repeated surveillance for thyroid nodules, the enduring uncertainty, and the absence of a clear treatment endpoint. Evans et al. found that the thyroid cancer-related fear scores among patients who had been diagnosed with benign nodules by FNA in the preceding 30 days were similar to those of actual thyroid cancer patients. Furthermore, when participants were asked to imagine their nodules as malignant and were shown cancer treatment-related videos, their anxiety scores increased significantly (Evans et al., 2024). These results suggest that substantial anxiety may remain even after a benign diagnosis, and concerns about potential malignancy may persist for some patients even after FNA and can be triggered under specific circumstances.

## 4 QOL and psychological status in low-risk DTC patients under active surveillance

An increasing number of studies have evaluated the long-term QOL and psychological status of patients with low-risk DTC undergoing active surveillance (AS) and surgery (OP) in recent years, and the findings consistently show that AS offers significant advantages over OP as far as the QOL is concerned (Yoon et al., 2024). In a prospective study conducted in China, Liu C. et al. (2024) utilized propensity-score matching to compare the QOL scores of 504 AS patients and 168 OP patients over median follow-up periods of 24 and 14.2 months, respectively. At follow-up initiation, AS patients reported significantly better physical function, role function, and social function, as well as global health status, compared to OP patients assessed 1 week after surgery. These superior QOL scores in the AS group persisted throughout the follow-up period, while most parameters remained stable over time. A multicenter prospective study conducted by Kong et al. (2019) in Korea reported similar findings, suggesting that AS provides better QOL both at the onset and during follow-up compared to surgical management.

Multiple studies have reported better symptom- and function-related outcomes in patients undergoing AS, which can be attributed to the fact that most of these patients are able to avoid surgery and the related adverse events. In a multicenter prospective cohort study conducted by Moon et al. (2021) in Korea (AS: *n* = 500 vs. OP: *n* = 317), the AS group had significantly better scores than the OP group in multiple domains, including voice changes, swallowing difficulty, fatigue, sleep changes and overall physical health, during 24 months of observation. Other similar prospective and retrospective studies have reported comparable findings (Kong et al., 2019; Jeon et al., 2019; Nakamura et al., 2020; Yoshida et al., 2020; Kazusaka et al., 2023; Chen et al., 2023; Kim M. J. et al., 2024). Furthermore, Liu C. et al. (2024) found that while voice and throat/mouth discomfort in the OP group gradually improved over time and reached levels similar to that of the AS group at 12 months post-operation, patients continued to report significantly worse outcomes for scar appearance, fatigue, loss of appetite and other parameters after undergoing surgery. In a prospective study conducted in Canada, Sawka et al. found that compared to the OP patients, AS patients had significantly better scores regarding the impact of disease symptoms interfering with daily life (Sawka et al., 2024).

Regarding psychological outcomes, current studies indicate that AS and OP patients have similar scores of common mental health parameters such as anxiety and depression, while AS leads to better scores in several certain psychological parameters. Liu C. et al. (2024) found that anxiety and depression scores of AS and OP groups were similar at baseline and during follow-up, and remained stable over time in both groups. Kong et al. (2019) reported consistent findings in a multicenter study, and further observed that AS group had significantly better outcomes in parameters such as coping with disease, change in self-concept, and feelings of isolation. Notably, a Japanese cross-sectional study involving patients with longer follow-up periods (AS: mean 56.5 months vs. OP: mean 84 months) reported significantly better anxiety and depression scores of AS patients compared to OP patients at the time of assessment (Nakamura et al., 2020). Current research suggests that the psychological benefits of AS are mainly pronounced when compared with total thyroidectomy (TT), whereas little or no difference is observed relative to hemithyroidectomy (HT) (Moon et al., 2021; Jeon et al., 2019; Nakamura et al., 2020). Some studies also indicate that anxiety levels among AS patients may be related to individual personality traits, and those exhibiting better trait anxiety experience a significant improvement in symptoms during long-term follow-up (Yoshida et al., 2020; Kazusaka et al., 2023).

Given that DTC patients undergo prolonged AS while harboring tumors, fear of progression is the most common psychological concern, though it tends to decrease over time. Sawka et al. reported a continuous decline in fear-of-progression scores among AS patients over 1 year of follow-up (Kim M. J. et al., 2024). Similarly, Davies et al. (2019) reported that among AS patients with less than 3 years of follow-up, 44% experienced recent worries about the cancer at least occasionally, 37% reported that these concerns had somewhat or greatly affected their mood, and 55% stated that such worries had lessened compared to the time of initial diagnosis; however, for those followed up for more than 3 years, the above percentages were 33%, 29%, and 63%, respectively (Sawka et al., 2024). It is noteworthy that fear of progression among AS patients does not appear to be worse than that observed for OP patients. Sawka et al. found that while AS patients had significantly better fear-of-progression scores compared to the OP group at baseline, the difference was no longer significant after 1 year of follow-up (Kim M. J. et al., 2024). Similarly, a cross-sectional study conducted by Jeon et al. (2019) on patients with longer follow-up durations (mean follow-up: 29.6 months for AS and 38 months for HT) reported no significant differences in fear-of-progression scores between the two groups at the time of assessment.

Nevertheless, some AS patients may discontinue surveillance and undergo surgery, not due to tumor progression but as a result of persistent fear, chronic anxiety, or other psychological stressors. Studies have shown that for these patients, surgery does not alleviate their psychological or physical burdens. Moon et al. reported that patients who changed their treatment choice from AS to surgery for reasons other than disease progression experienced declines in physical, psychological, social and overall health scores post-operation, and the physical scores were significantly lower than that of patients who underwent surgery due to disease progression. In contrast, among patients who crossed over to surgery due to disease progression, most QOL parameters remained stable before and after surgery (Moon et al., 2021). Sawka et al. found no significant difference in decision regret scores between patients who remained on AS and those who underwent OP initially; however, patients who crossed over from AS to OP had significantly higher decision regret scores, indicating greater dissatisfaction and higher level of regret (Kim M. J. et al., 2024).

Psychological and emotional factors not only influence changes in treatment strategy during AS, but also play a pivotal role in the initial treatment decision-making process. Multiple studies have reported that negative emotions in patients reduce their acceptance of AS and increase the preference for surgery (Pitt et al., 2021b,a; Jensen et al., 2020). In the study by Zhu et al., the vast majority of patients who had explicitly declined AS stated that psychological distress and negative emotions associated with the cancer were the primary reasons for choosing surgery. Some even acknowledged that the persistent awareness of having the disease and living with the tumor were intolerable. Conversely, over half of the patients who chose AS indicated that a positive and optimistic attitude was a key facilitator of their decision (Zhu et al., 2023b). Notably, the final treatment decision of the patients is influenced not only by psychological and emotional factors, but also their understanding of the disease and treatment options, access to medical resources, limitations in AS follow-up conditions, recommendations of attending physicians, and socioeconomic status (D'Agostino et al., 2018; Nickel et al., 2018; Sawka et al., 2022).

Thermal ablation techniques are increasingly being applied for patients with low-risk DTC. However, compared to conventional surgery and AS, there are concerns regarding the long-term efficacy, appropriate indications, and clinical positioning of thermal ablation. Current evidence suggests that due to the avoidance of surgery-related adverse events, most patients who choose thermal ablation report better QOL and psychological scores compared with those undergo surgery (Zheng et al., 2023; Kong et al., 2024). Moreover, unlike AS, thermal ablation offers a relatively clear treatment intervention and endpoint, which may provide notable psychological advantages to patients. In a prospective study of 227 patients with papillary thyroid microcarcinoma (PTMC), approximately 40% of the patients who initially chose AS opted for radiofrequency ablation (RFA) eventually, while none opted for surgery after RFA due to psychological reasons (Lee et al., 2024). However, the QOL and psychological status of patients who experience recurrence or residual disease after thermal ablation have not been sufficiently evaluated. Furthermore, no study so far has directly compared the QOL and psychological outcomes following thermal ablation, AS, and surgery.

## 5 QOL and psychological status of DTC patients undergoing standard treatment

### 5.1 Impact of surgery on QOL and psychological status

Surgery remains the primary treatment for most DTC patients. While surgical intervention is effective for tumor removal, it is also associated with various complications, with hypoparathyroidism being one of the most common postoperative issues. Studies show that the post-surgery incidence rates of temporary and permanent hypoparathyroidism can reach up to 38% and 3%, respectively (Edafe et al., 2014). Hypoparathyroidism can cause a wide range of hypocalcemia-related symptoms, such as tingling or numbness in the fingers and feet, muscle cramps, fatigue, and difficulty concentrating. Patients with hypoparathyroidism generally experience impaired QOL, and greater symptom burden results in more pronounced impairment (Hadker et al., 2014; Büttner et al., 2022). In fact, most hypoparathyroidism patients exhibit significantly lower scores in at least one QOL domain compared to healthy population or controls (Büttner et al., 2024). A retrospective study of thyroid cancer patients showed that those with postoperative hypoparathyroidism had significantly worse QOL scores compared to those without, in addition, hypoparathyroidism showed an independent negative correlation with multiple QOL domains (Büttner et al., 2020). Beyond persistent symptoms, the need for lifelong, multiple daily doses of calcium and vitamin D supplements, as well as the fear of recurring hypocalcemia symptoms can further increase psychological stress and trigger anxiety or depression. Missaoui et al. (2022) reported that permanent hypoparathyroidism after DTC surgery significantly worsens patient QOL, particularly affecting mental health. Noto et al. (2022) also found that permanent hypoparathyroidism was significantly associated with both anxiety and depression in DTC patients.

Recurrent laryngeal nerve (RLN) injury is another common and serious complication of thyroid surgery. Although the overall risk is relatively lower, the incidence of temporary and permanent RLN paralysis following surgery can reach 3.9% and 1.6%, respectively (Wong et al., 2017). RLN injury may lead to hoarseness, swallowing difficulties, and breathing problems, which can seriously affect the daily functioning and mental health of patients (Noto et al., 2022; Jones et al., 2018). Notably, the number of patients reporting voice or swallowing discomfort after surgery is much higher than the actual incidence of RLN injury. In a survey of 1,743 thyroid cancer patients, 63% reported dysphagia and 71% reported dysphonia, which were the most common short-term adverse effects after surgery (Goswami et al., 2019). Even among patients without vocal fold paralysis or immobility confirmed by laryngoscopy, 50% still reported voice problems 1 year after surgery (Kletzien et al., 2018). Difficulty in swallowing and voice changes are significantly associated with worse scores in physical and social functions, as well as higher levels of fatigue, pain, anxiety, and depression (Goswami et al., 2019). These findings highlight that voice and swallowing problems after thyroid surgery can substantially diminish both QOL and psychological health.

Since the risk of surgical complications is closely related to the extent of surgery, different surgical extent can have varying impacts on the patients' QOL. This pattern is similar for both low-risk and non-low-risk DTC patients. A recent systematic review of DTC patients showed that hemithyroidectomy (HT) generally results in better QOL outcomes than total thyroidectomy (TT), particularly in physical domains (such as voice, fatigue, appetite, and scarring) and social domains (including social function, role function, social support, and impact at work) (Landry et al., 2022). Another systematic review focusing specifically on low-risk DTC patients reported similar results (Bach et al., 2024). However, these advantages may diminish over time. In a prospective study conducted by Chen et al., patients who underwent HT had significantly better QOL scores compared to those who underwent TT during the first 1–3 months after surgery, but the differences were no longer significant at 6–12 months post-operation. A similar trend was also observed for psychological wellbeing; both anxiety and depression scores were significantly better in the HT group 1 month after surgery, but the differences disappeared by 3–6 months post-operation (Chen et al., 2022). Several long-term prospective cohort and cross-sectional studies have reported consistent results (Bongers et al., 2020; Yang et al., 2021; van Gerwen et al., 2022). Furthermore, most studies have found no significant difference in the level of disease-related fear between HT and TT groups (Landry et al., 2022; Lan et al., 2021; Lee et al., 2025). However, there are reports suggesting that fear of recurrence tends to be higher in patients who undergo HT compared to those who undergo TT (Bongers et al., 2020), and that concerns regarding recurrence increases over time among HT patients and decreases among TT patients (Antunez et al., 2024). Currently, there is a lack of high-quality evidence regarding the relationship between surgical extent and disease-related fear, and further research is needed to clarify this issue.

In addition to surgical complications, scarring and related discomfort in the anterior neck following conventional open thyroidectomy (OT) are significant sources of distress for patients. Studies have demonstrated a negative correlation between patient-reported scar appearance and QOL, particularly in the domains of emotional function (e.g., reduced self-esteem, feelings of helplessness, isolation) and social function (e.g., avoidance of social interactions, restricted choice of employment) (Liu et al., 2021; Zhu et al., 2023a). Some studies have even reported that QOL scores among OT patients with visible neck scars are comparable to those of individuals with psoriasis or severe atopic dermatitis, and that patients with scar-related symptoms (e.g., itching, pain, burning, tightness) report significantly worse QOL outcomes than those without such symptoms (Choi et al., 2014; Arora et al., 2016). Notably, concerns about scar appearance may gradually improve over time (Bach et al., 2022; Kurumety et al., 2019), although this trend is not consistently observed across all studies (Liu C. et al., 2024). Such variability may be influenced by factors such as gender, age, ethnicity, and cultural background (Zhu et al., 2023a; Bach et al., 2022; Kurumety et al., 2019). For instance, a survey conducted among Turkish and Korean populations found that young Korean women were particularly affected by visible thyroidectomy scars (Alci et al., 2023).

Nevertheless, issues related to scarring have improved significantly with increasing use of endoscopic (ET) and robotic (RT) thyroidectomy. Multiple studies have demonstrated that, regardless of the surgical approach (transaxillary, transoral, or other approaches), patients undergoing ET/RT report significantly better long-term satisfaction with scar appearance compared with those undergoing OT (Huang et al., 2016; Wu et al., 2025; Johri et al., 2020; Lee et al., 2014), and the differences between various minimally invasive approaches are negligible (Nguyen et al., 2024). The QOL advantages of ET/RT may be most apparent in the early postoperative period. Multiple prospective and retrospective studies with follow-up periods within 12 months have consistently shown that ET/RT patients have significantly better scores across multiple QOL domains compared to OT patients (Nguyen et al., 2024; Zhou et al., 2024). However, a few studies have reported less favorable outcomes in terms of pain, neck discomfort and physical symptoms with the transoral approach (Wu et al., 2025; Zhou et al., 2024; Ha et al., 2018; Deshmukh et al., 2024). A retrospective study showed that patients who underwent transoral ET via the vestibular approach had better scar satisfaction than those in the OT group, and swallowing, voice, physical function, or pain were similar in both groups over a median follow-up period of 35 months (Nagururu et al., 2024). Furthermore, while anxiety and depression tend to improve over time after ET/RT as well as OT, studies have not identified significant differences in these psychological outcomes between the two groups (Huang et al., 2016; Wu et al., 2025; Johri et al., 2020; Kasemsiri et al., 2020). These findings suggest that scarring may not be the sole determinant of postoperative psychological health, and concerns such as fear of recurrence may play a more important role.

Recently, Kim et al. conducted a comprehensive 5-year prospective study in Korea to evaluate longitudinal changes in QOL among 185 DTC patients before and after thyroidectomy. Most QOL domains scores declined significantly at 3 months post-operation compared to the preoperative baseline. While most parameters such as pain, swallowing, and physical function gradually improved and returned to baseline levels within 6–12 months after surgery, certain parameters such as voice changes and cosmetic appearance did not fully recover even during long-term follow-up. Psychological health parameters such as anxiety, depression, fear of tests, and fear of recurrence improved significantly within 3–6 months post-operation and were sustained throughout the 5-year follow-up period (Kim B. H. et al., 2024). These changes in QOL and psychological health were influenced by multiple factors, such as surgical approach, surgical complications, and extent of surgery, as well as subsequent treatments including radioactive iodine (RAI) therapy and thyroid hormone therapy, each exerting varying degrees of impact.

### 5.2 Impact of RAI therapy on QOL and psychological status

RAI scanning and therapy are important adjuncts in the management of DTC. Studies show that over 50% of DTC patients experience significant anxiety and fear prior to RAI, with levels significantly higher than those observed in patients not requiring RAI or among healthy controls (Barbus et al., 2018; Changchien et al., 2024). Factors contributing to this emotional response include female gender and insufficient understanding of RAI. Moreover, patients undergoing repeated RAI treatments may experience heightened pre-RAI anxiety due to previous negative experiences (Qiao et al., 2022). Additionally, hypothyroidism induced by thyroid hormone withdrawal (THW) prior to RAI is a major factor contributing to diminished QOL and psychological health. Chow et al. (2006) reported that most QOL scores remained stable at 2 weeks after THW compared to the baseline levels; however, by the fourth week, patients reported significantly worse scores in physical, social, emotional, and overall QOL domains, indicating a progressively negative impact with prolonged THW duration. In contrast, recombinant human thyroid-stimulating hormone (rhTSH), which prevents the onset of hypothyroidism and its associated adverse effects, has demonstrated clear advantages over THW in preserving patient QOL (Lee et al., 2010). A placebo-controlled, double-blinded and randomized crossover clinical trial (*n* = 56) showed that patients treated with rhTSH had significantly better scores for general health, social functioning, mental health, physical symptoms, and psychological symptoms compared to patients in the THW group (Nygaard et al., 2013). Additionally, since patients' bodies (especially urine, saliva, and sweat) remain radioactive for several days after treatment, social isolation is typically required. This period of social isolation may be particularly difficult for patients who are already feeling vulnerable. Studies report that about 51.9% of patients experience anxiety about isolation prior to RAI, which is positively correlated with fear of the treatment itself (Barbus et al., 2018).

The adverse effects of RAI therapy can significantly affect both QOL and psychological health. Lubitz et al. reported that among 1,167 DTC patients who received RAI, 53.2% experienced symptoms such as dry mouth, dry or watery eyes, and pain with eating (Lubitz et al., 2021). Ming et al. further demonstrated that DTC patients had significantly worse scores in domains including dry mouth, gastrointestinal disturbance, insomnia, role functioning, and social functioning at 3 days post-RAI compared to the pre-treatment levels. Most of these issues did not improve after 1 month, and some symptoms such as fatigue, pain, joint discomfort, fear and aspect of physical health even worsened over time (Ming et al., 2022). Moreover, physical pain and social function were reported to be significantly correlated with anxiety and depression (Changchien et al., 2024). Some studies have also reported a positive dose-response relationship between physical functioning and RAI dosage, although there is currently no consensus regarding the association between RAI dosage and adverse effects (Ming et al., 2022; Dingle et al., 2013; Legrand et al., 2024). It is important to note that many DTC patients are women of reproductive age (20–40 years). For those planning pregnancy, patients are often advised to delay pregnancy after RAI therapy. Studies show that the median time from diagnosis to first childbirth is significantly longer in patients who undergo RAI compared to those who do not (34.5 vs. 26.1 months, *p* < 0.001), with delays observed across all age groups from 20 to 39 years (Wu et al., 2015). Among reproductive-age women, delayed childbearing is closely linked to psychological distress, anxiety, and depression (Wasser et al., 2024). However, the specific psychological impact of RAI-related fertility delays in this population warrants further study.

As with surgery, the negative effects of RAI therapy tend to improve over time. Hsieh et al. observed that pain, anxiety, depression, and both physical and mental health scores significantly improved within 6–12 months after RAI. Furthermore, the scores of pain and physical health returned to baseline levels by 12 months, while mental health scores surpassed pre-treatment levels (Hsieh et al., 2024). Other studies with follow-up periods within 1 year have reported comparable results (Banihashem et al., 2020; Wu et al., 2016). A cross-sectional survey comparing 100 matched pairs of RAI-treated and control (non-RAI) post-TT patients (mean follow-up: 3.5 years for the RAI group and 3.8 years for the non-RAI group) showed no significant differences between the two groups in most QOL domains, including physical functioning, role-physical, social functioning, throat/mouth symptoms, voice, mental health, and fear of progression (Ahn et al., 2020).

### 5.3 Impact of TSH suppression therapy on QOL and psychological status

Some high-risk DTC patients require higher-than-replacement doses of thyroid hormone to suppress TSH levels, thereby inducing a state of subclinical or mild hyperthyroidism to reduce the risk of recurrence. However, its effects on QOL and mental health remain inconclusive. Studies suggest that long-term TSH suppression and supraphysiologic doses of thyroid hormone may result in symptoms such as palpitations, atrial fibrillation, anxiety, and impaired concentration, as well as increased risk of cardiovascular disease and osteoporosis (Gubbi et al., 2024). Braafladt et al. (2025) reported that DTC patients who received higher defined daily doses (DDD) of levothyroxine post-operation had significantly lower QOL scores, particularly in the physical, social, and mental health domains, which was indicative of a dose-dependent relationship. Similarly, a questionnaire-based study involving 2,584 DTC patients showed that TSH suppression therapy was significantly associated with reduced energy and increased fatigue 2–4 years after surgery (Hughes et al., 2020). A recent study also found that in DTC patients receiving TSH suppression therapy, lower TSH levels were significantly associated with poorer mental health and social functioning scores (Missaoui et al., 2022). In addition, studies show that variations in TSH levels (particularly lower levels or a downward trend) correlate significantly with higher risks of depression, anxiety, cognitive problems, and various psychiatric disorders (e.g., suicidal ideation), potentially involving neuroendocrine regulation, metabolic disturbances, or autoimmune mechanisms (Chen et al., 2024; Deng et al., 2025).

In contrast, thyroid hormone replacement therapy aims to maintain TSH within the normal range, using lower doses that generally do not cause hyperthyroidism or related impairments in QOL or mental health. However, some long-term follow-up studies have not observed declines in quality of life or psychological disorders in patients receiving either TSH replacement or suppression therapy. For instance, a prospective Swedish study conducted by Winter et al. (*n* = 351, 5-year follow-up) did not demonstrate any significant correlation between TSH levels and physical component summary score (*p* = 0.499) or mental component summary score (*p* = 0.797). Additionally, TSH levels were also not predictive of changes in these scores over time (*p* = 0.438, 0.590), and no significant differences were observed among patient groups stratified by the extent of TSH suppression at the fifth year of follow-up (Winter et al., 2024). Similarly, Monzani et al. (2023) found that patients with complete TSH suppression had similar cores of anxiety, depression, and symptoms of hypo- or hyperthyroidism compared to those with mild TSH suppression or hormone replacement therapy; a significant difference was observed only in the “overall impact” parameters when compared to the TSH replacement group (*p* = 0.036). In addition, Kim B. H. et al. (2024) found that hormone replacement had no significant impact on QOL or psychological scores compared to the control (non-replacement) group among post-HT patients. A prospective, single-blind, randomized clinical trial involving DTC patients who had received TSH suppression therapy for more than 10 years after TT showed no significant differences in the baseline QOL or psychological health scores between the continuous levothyroxine group (low TSH) or levothyroxine plus placebo (restored normal TSH) group under comparable medication frequency and treatment durations. In addition, most scores also remained unchanged from baseline to 6 months post-intervention in both groups (Eustatia-Rutten et al., 2006).

High-quality evidence regarding the impact of TSH suppression or replacement therapy on QOL and mental health of DTC patients remains limited, and the published findings are inconsistent. These discrepancies are largely attributed to methodological challenges encountered in the design and implementation of these studies. First, grouping patients by TSH suppression or replacement status, whether by presence or absence of therapy, TSH levels, or thyroid hormone dosage often fails to accurately reflect actual hormonal exposure, and is affected by individual variations and measurement errors. Second, TSH replacement or suppression therapy is typically a long-term treatment process, during which the patients' QOL and psychological health are influenced by multiple factors, including the extent of surgery, surgical trauma and complications, iodine-131 therapy, fear of recurrence, and emotional fluctuations. Thus, isolating the independent effects of TSH suppression or replacement therapy is challenging. Future research should focus on conducting multi-level, high-quality studies tailored to patient conditions, extent of surgery, treatment stages, and medication regimens to clarify the true effects of TSH suppression or replacement therapy.

## 6 QOL and psychological status of DTC patients during long-term follow-up

Recent advances in medical philosophy and technology have led to a paradigm shift in the management of DTC—from an exclusive emphasis on oncologic safety to a more comprehensive approach that takes into account the long-term QOL and psychological wellbeing of the patients. In this context, diagnostic and therapeutic strategies are continually being optimized to minimize overtreatment and treatment-related adverse events. Although many of the physical and psychological adverse effects experienced by DTC patients throughout the clinical care continuum may improve over time, the overall QOL and mental health of survivors remain significantly lower than that of the healthy population (Monzani et al., 2023; Goswami et al., 2018). Furthermore, anxiety, depression, fatigue, and sleep disturbances may even be worse for DTC survivors compared to that reported by survivors of other cancers with poorer prognoses, including non-Hodgkin lymphoma, breast cancer, colorectal cancer, and uterine cancer (Goswami et al., 2018).

This disparity may be related to the unique circumstances of DTC patients, who experience long-term survival while coping with the psychological impact of a cancer diagnosis and the enduring fear of disease recurrence. Studies show that fear of recurrence is extremely common among DTC survivors (Hampton et al., 2024), and significantly correlates with worse outcomes across nearly all measures of QOL and mental health (Hedman et al., 2018). Routine follow-up visits and examinations may trigger concerns about recurrence, causing patients to experience ongoing cycles of anxiety and distress throughout their survivorship (Hedman et al., 2017). Furthermore, studies indicate that DTC patients often lack adequate information about their disease, treatment-related adverse effects, long-term management strategies, and access to professional psychological support (Das et al., 2025). Some patients also report that the perception of thyroid cancer being a “good cancer” can hinder their efforts to seek information and psychological assistance, thereby exacerbating feelings of isolation and helplessness (Hyun et al., 2016). Additionally, challenges such as declining treatment adherence over time, the management of new or chronic comorbidities, and caregiver stress can further compromise patients' overall health (Mongelli et al., 2020; O'Neill et al., 2023). These adverse factors can further accentuate the QOL and mental health issues among DTC patients, a population characterized by both high incidence rates and high survival rates.

## 7 Clinical implications

**Figure 1:**
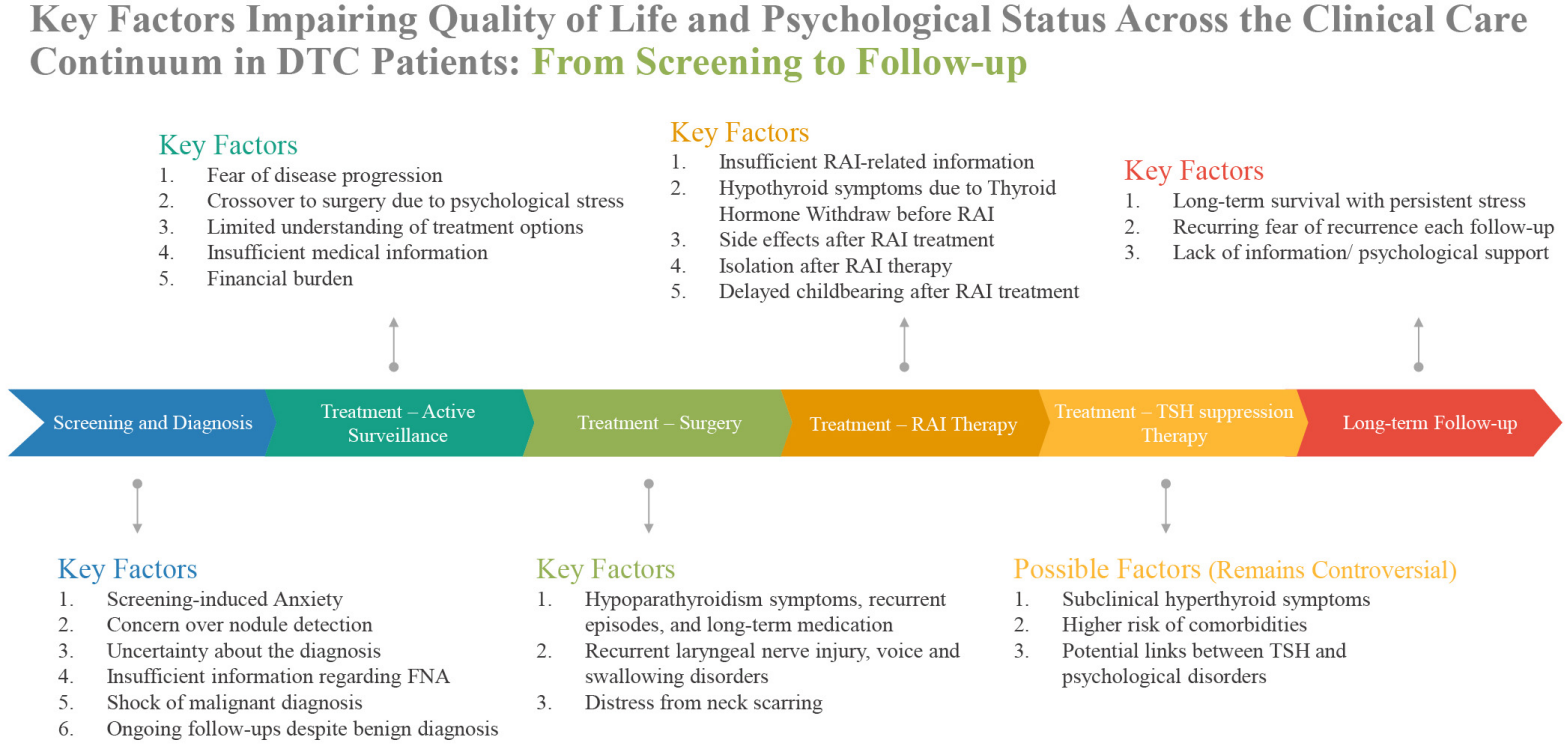
Key factors impairing quality of life and psychological status across the clinical care continuum in DTC patients: from screening to follow-up.

Despite the generally favorable prognosis, DTC patients frequently experience significant impairments in quality of life and psychological wellbeing throughout the entire clinical care continuum, from screening and diagnosis to treatment and long-term follow-up (Figure 1). These findings suggest that routine clinical care for DTC patients should not only focus on oncological outcomes, but also on the assessment and management of psychological distress, fear of recurrence, and treatment-related adverse effects. Multidisciplinary interventions—including psychological counseling, patient education, and long-term support systems—are essential to address these issues and improve patient outcomes. Integrating mental health support and quality of life assessments into standard DTC management strategy can enhance patient satisfaction, treatment adherence, and overall survival with high quality of life.

## 8 Limitations

This narrative review has several limitations. First, the included literature is subject to publication bias and heterogeneity in study design, populations, and assessment tools. Second, most available studies are observational or cross-sectional, with a lack of high-quality interventional research specifically focusing on the psychological and quality of life outcomes in DTC patients. Future research should focus on prospective, multi-center, and interventional studies with standardized tools to better clarify the unique challenges of DTC patients and to evaluated the effectiveness of targeted interventions.

## 9 Conclusion

In conclusion, DTC patients commonly experience impaired QOL and psychological distress across all stages of clinical care. In the future, it will be essential to further optimize the management of DTC patients throughout the clinical care continuum by establishing effective informational and psychological support systems, and developing standardized, evidence-based interventions to address the needs of patients at each stage of care. These measures can not only improve therapeutic efficacy, but also enhance the overall QOL and psychological wellbeing of DTC patients, promoting long-term, high-quality survival comparable to that of the general population.
